# A single case report of STN-DBS for severe crack-cocaine dependence: double-blind ON vs. SHAM randomized controlled assessment

**DOI:** 10.3389/fpsyt.2023.1146492

**Published:** 2023-05-25

**Authors:** Florence Vorspan, Philippe Domenech, David Grabli, Jérôme Yelnik, Marine Delavest, Charles Dauré, Frank Bellivier, Antoine Pelissolo, Hayat Belaid, Christelle Baunez, Carine Karachi, Luc Mallet

**Affiliations:** ^1^Université de Paris Cité, INSERM UMRS 1144, Paris, Île-de-France, France; ^2^Assistance Publique - Hôpitaux de Paris, GHU NORD, GH Lariboisière-Fernand Widal, Département de Psychiatrie et de Médecine Addictologique, Paris, Île-de-France, France; ^3^Sorbonne Université, Institut du Cerveau - Paris Brain Institute - ICM, INSERM U1127, CNRS UMR 7225, Paris, Île-de-France, France; ^4^Assistance Publique-Hôpitaux de Paris, Hôpitaux Universitaires Henri Mondor - Albert Chenevier, DMU IMPACT, Département Médical-Universitaire de Psychiatrie et d'Addictologie, Créteil, France; ^5^Assistance Publique - Hôpitaux de Paris, GHU Sorbonne Université, Hôpital Pitié-Salpêtrière, Département de Neurologie, Paris, Île-de-France, France; ^6^Université Paris-Est Créteil, Créteil, Ile-de-France, France; ^7^Assistance Publique - Hôpitaux de Paris, GHU Sorbonne Université, Hôpital Pitié-Salpêtrière, Service de Neurochirurgie, Paris, Île-de-France, France; ^8^UMR7289 CNRS & Aix-Marseille Université, Marseille, Provence-Alpes-Côôte-d'Azur, France; ^9^Department of Mental Health and Psychiatry, Global Health Institute, University of Geneva, Geneva, Switzerland

**Keywords:** cocaine dependence, deep brain stimulation, subthalamic nucleus, crack-cocaine, controlled clinical trial

## Abstract

Crack-cocaine dependence is a severe condition with a high mortality rate. This single case study report details the first deep brain stimulation (DBS) trial targeting the sub-thalamic nucleus (STN) for crack-cocaine dependence. The investigation aimed to assess the effects of STN-DBS on cocaine craving and cocaine use, as well as STN-DBS safety and tolerance in this indication. In this pilot study, we performed double blind cross-over trials, with “ON-DBS” vs. “SHAM-DBS” for 1-month periods. STN-DBS failed to reduce cocaine craving and use. An episode of DBS-induced hypomania occurred after several weeks of cocaine intake at stimulation parameters previously well tolerated. Future research on cocaine dependence should be conducted after a prolonged abstinence period and/or explore novel types of stimulation patterns.

## Introduction

Crack cocaine dependence increases mortality (1, 2) and has significant health and social consequences (3–5). France is facing an increase in specialized addiction treatment entry for cocaine use disorder (6) in general, and an increase in crack-cocaine related mortality specifically (7). Current treatments (8, 9) show limited efficacy on abstinence maintenance and harm reduction. To date, four reported clinical cases of cocaine or methamphetamine addiction have utilized deep brain stimulation (DBS) treatment, with 3 out 4 patients showing a reduction in cocaine use (10–12), reviewed in (13). These trial studies targeted the bilateral ventral striatum, with parameters between 150–165 Hz, 2–3.3 V and 150–240 μs, with 240 μs in two cases associated with the emergence of hypomania. Three of the four trials were open label (10, 12), whilst the one double blind trial failed to show any difference between the ON vs. SHAM period (11). Consequently, causal modeling trial efficacy is subject to influence by non-specific factors.

We previously advocated the subthalamic nucleus (STN) as a potential target for severe cocaine dependence (14). Preclinical studies indicate that the STN is an important hub for controlling cocaine intake. High frequency STN-DBS corrects the balance between sucrose and cocaine preference in a conditioned place paradigm (15) and reduces re-escalation of cocaine self-administration after prolonged, but not short, abstinence (16). Low frequency STN-DBS also reduces cocaine intake in cocaine-dependent rats who developed an aversive shock-resistant cocaine intake (17). In humans, STN-DBS combined with a decrease of dopaminergic medication has been proposed to improve both medication abuse and other behavioral addictions in Parkinson's disease (PD) patients (18). STN-DBS also improves obsessions and compulsions in obsessive-compulsive disorder (OCD) and PD patients (19–22). The core cocaine dependence symptom, craving, has strong parallels with obsessional symptoms in OCD (23). There is also a high prevalence of transient OCD-like symptoms in patients with cocaine use disorder in care settings, with an OCD-like symptom prevalence of 58% in one study (24), mostly consisting of checking, and repetitive/ritualized movements. Within the general population, cocaine use positively associates with OCD with an odd-ratio of 4 (25). Thus, our hypothesis is that STN-DBS suppresses craving during abstinence, as indicated by the Obsessive Compulsive Cocaine Score (OCCS) score (26), in people with cocaine addiction.

## Method

We present the first STN-DBS case for severe and treatment-resistant crack cocaine use disorder over a 2-year follow-up. The study (Clinicaltrials.gov Number NCT02892851) was approved by a local ethics committee. Inclusion criteria were diagnosis of DSM IV cocaine dependence; crack cocaine use (a smoked form of cocaine); previously classed as non-responder to a well conducted pharmacological treatment, with one long-term detoxification stay associated with after-care. Exclusion criteria were limited to surgery contra-indications or unstable psychiatric condition. Consequently, all stable psychiatric conditions and other substance use disorders were not subject to exclusion. The primary outcome was OCCS reduction (26), secondary outcomes were crack cocaine scale (CCQ) (27) immediate craving score reduction and self-declared and objective cocaine use reduction over the cross-over 1 months periods. One male patient in his 40 s with a 20-year history of severe smoked crack dependence participated in this trial. He was also opioid and benzodiazepine dependent, as well as satisfying criteria for DSM IV alcohol abuse. He was unemployed and homeless, with severed family ties for 11 years. The study participant had 2 previous in-patient cocaine cessation trials, followed by long-term residential care with a maximum abstinence period of 4 months, followed by relapses. He had also received 2 months of aripiprazole up to 15 mg/d (19) with no efficacy. Finally, he failed to attend cognitive behavioral therapy (CBT) relapse prevention group sessions. At inclusion, he was under prescribed oral buprenorphine 8 mg/day, cyamemazine 300 mg/d and oxazepam 150 mg/day.

## Results

During the hospitalization for pre-surgical check-ups and 2 days before the scheduled surgery date, the patient discharged himself and relapsed. Nine days later, he presented himself to the outpatient facility asking to be “re-enrolled” and gave a new written consent a few days later. The OCCS measurement of craving before surgery was 50/56.

**Figure 1:**
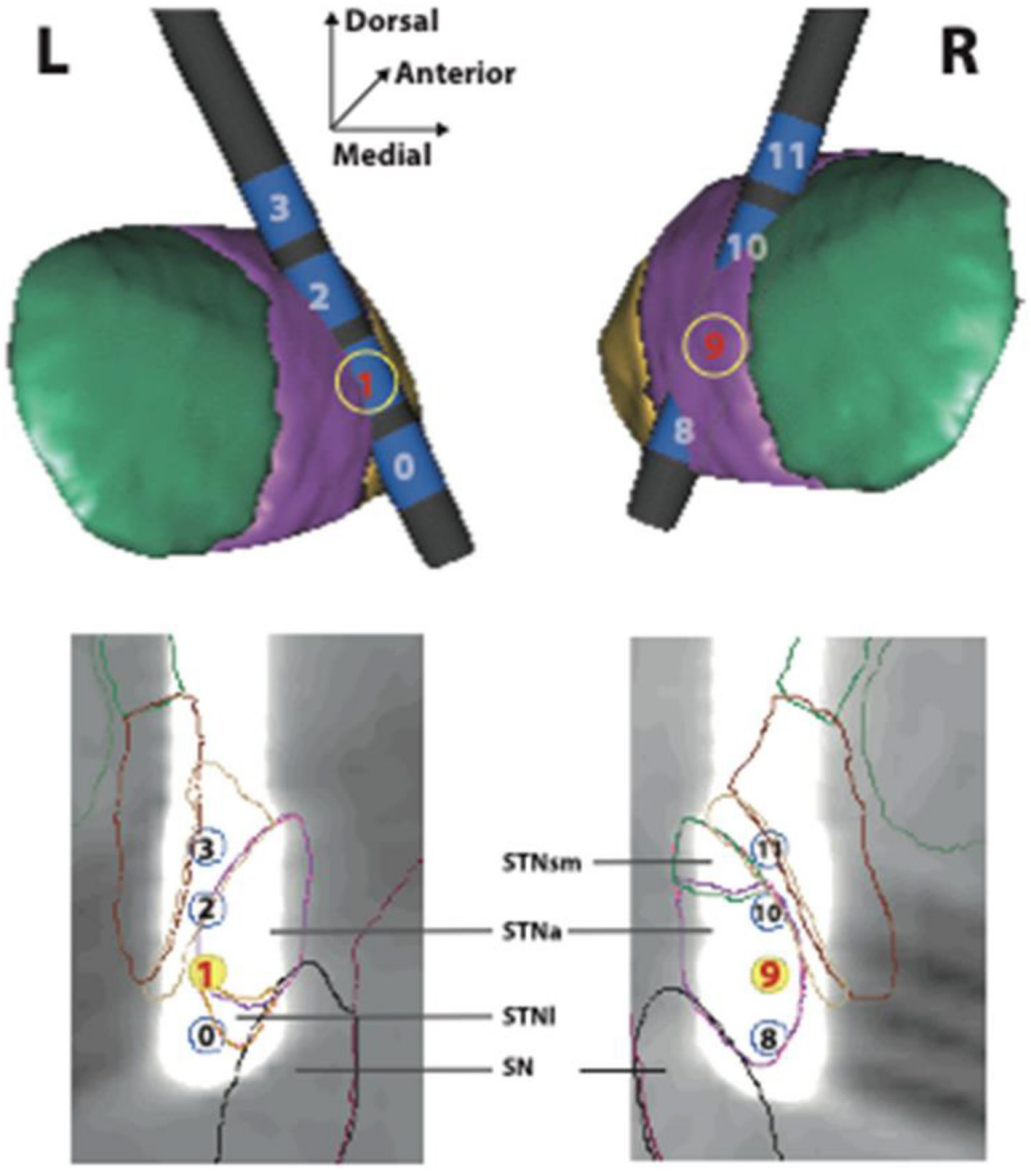
Location of stimulating electrodes in the STN region. Localization of stimulating electrodes. (Upper frame) The left (L) and right (R) subthalamic nuclei are seen from a posterior view on 3D reconstructions from a computerized atlas (24). The sensori-motor (green), associative (purple) and limbic (yellow) subdivisions are shown. The individual electrode contacts (in blue) are numbered (0–3 and 8–11). (Lower frame) The artifact produced by each electrode in the MRI acquisition is shown in a plane of section parallel to its long axis. The exact localization of each contact is indicated in relation to the sensori-motor, associative, limbic subdivisions of the subthalamic nucleus in this plane (STNsm, STNa, STNl). The substantia nigra is also shown (SN).

STN-DBS surgery was performed 3 months later. Bilateral electrodes (Medtronic, Minneapolis, 3389 connected to an Activa PC generator) were implanted under general anesthesia. Parameter testing revealed stimulation-induced diplopia on both ventral contacts (0 and 8; >3 V, 60 μs, 130 Hz, all impedances < 2,000 Ω), as well as mild hypomania after 3 h of unipolar stimulation on contact 9 (2.5 V) and dyskinesia/akathisia after 4 h of unipolar stimulation on contact 1 (2.5 V), but not on contact 0 or 2 [see electrode locations (28) in Figure 1].

The double-blind cross-over trial (1 month SHAM, then 1 month ON-DBS) started 1-month post-surgery. During the ON-DBS period, a bilateral unipolar stimulation on contact 1 and 9 at 2 V (60 μs, 130 Hz) was utilized, which was well tolerated. Treatment efficacy on craving could not be assessed due to absence of craving at the baseline (OCCS baseline: 0/56) (see Table 1) and remained low most of the time during this first double-blind cross-over.

**Table 1:** Primary and secondary outcomes of STN-DBS in this clinical case.

**Pre-inclusion September 2015 (outpatient)**	OCCS 50/56 (primary outcome) CCQ brief 16/70 Previous 4 weeks urinary cocaine tests [+/+/+/-] Self-declared use collected on the assessment day regarding previous week: 3–7 use per day; 3–7 days per week. Current treatment (mg/day): buprenorphine 8, oxazepam 100, cyamemazine 100, zopiclone 7.5				
**Randomization**	**4 weeks SHAM**		**2 weeks WASH-OUT**	**4 weeks ON Contacts 0 and 8;** >**3 V, 60** μ**s, 130 Hz**	
	**Baseline**	**End**		**Baseline**	**End**
CROSS-OVER 1 January-April 2016 Weekly urinary cocaine tests Self-declared use	OCCS 0/56 CCQ brief 10/70 [-/-/-/-] 0 use per day 0 day per week	OCCS 14/56 CCQ brief10/70 [?/?/?/-] 0 use per day 0 day per week		**OCCS 0/56 CCQ brief 10/70** [?/-/?/-] 0 use per day 0 day per week	**OCCS 2/56 CCQ brief 10/70** [-/-/?/+] 1 use per day 1 day per week
	Buprenorphine 8, paroxetine 20, oxazepam 80, cyamemazine 80, zopiclone 7.5			Buprenorphine 8, paroxetine 20, diazepam 50, cyamemazine 75, zopiclone 7.5	
**Full inpatient stay, open ward**					
**Randomization**	**4 weeks ON Contacts 0 and 8;** >**3 V, 60** μ**s, 130 Hz**		**2 weeks WASH-OUT**	**4 weeks SHAM**	
	**Baseline**	**End**		**Baseline**	**End**
CROSS-OVER 2 November 2016-January 2017 Weekly urinary cocaine tests Self-declared use	**OCCS 15/56 CCQ brief 14/70** [+/+/-/+] 1 per day 1 day per week	**Interrupted at day 1 Serious adverse effect Hypomania**	**-**	**-**	
	Buprenorphine 8 mg, paroxetine 60, diazepam 20, cyamemazine 50, zopiclone 7.5				
**Outpatient in social housing except for the assessment day, where inpatient in open ward**					
**Randomization**	**4 weeks SHAM**		**2 weeks WASH-OUT**	**4 weeks ON Contacts 1 and 9; 1.25 V, 60** μ**s, 130 Hz**	
	**Baseline**	**End**		**Baseline**	**End**
CROSS-OVER 3 February- May 2017 Weekly urinary cocaine tests Self-declared use	OCCS 36/56 CCQ brief 9/70 [+/+/+/+] 1–2 use per day 6–7 days per week	OCCS 3/56 CCQ brief 25/70 [-/+/+/-] 0 use per day 0 use per week		**OCCS 1/56 CCQ brief 10/70** [+/-/-/+] 0 use per day 0 use per week	**OCCS 30/56 CCQ brief 14/70** [-/+/+/+] 1 use per day 2–3 days per week
	Buprenorphine 8, paroxetine 60, diazepam 15, aripiprazole 20, topiramate 25, zopiclone 7.5			Buprenorphine 8, paroxetine 60, diazepam 15, aripiprazole 20, topiramate 25, zopiclone 7.5	
**Outpatient in social housing except for the five first days of each period, with a careful increase of the stimulation parameters in a closed ward**					
Last observation November 2017, OFF stimulation, outpatient OCCS 02/56 CCQ brief 10/70 Previous 4 weeks urinary cocaine tests [-/-/-/-] Self-declared use collected on the assessment day regarding the previous week: 0 use per day; 0 day per week. Current treatment (mg/day): paroxetine 60, aripiprazole 20					

OCCS, obsessive compulsive cocaine scale; CCQ, cocaine craving scale; +, positive cocaine screening; –, negative cocaine screening.

Because weekly urinary screening was only partially collected, a second crossover was decided and randomized (1 month ON-DBS, then 1 month SHAM). During this second cross-over attempt, the patient lived in a social housing facility and actively smoked crack (declared use 100 mg/day, 3 positive urinary tests in a month), so that he entered the ON-DBS phase with only two weekly urine cocaine free tests. After 12 h ON-DBS, following the same parameter setting protocol as previous, the patient exhibited an unexpected hypomanic episode, became unruly, and left before being brought by the police to the emergency department. When the DBS was turned off the following day, the hypomanic state quickly faded.

Finally, in accordance with the independent safety committee recommendations, and in agreement with the patient who believed that this state was an unprecedent feeling of wellbeing that could be useful to maintain crack abstinence, a third cross-over was initiated (1 month SHAM, then 1 month DBS-ON) with a progressive increase of DBS intensity over 5 days up to a reduced target intensity (contact 1 and 9; 1.25 V, 60 μs, 130 Hz) and performed in a closed ward (see Table 1). No hypomanic state occurred. The patient was discharged with the DBS ON.

During this second cross-over, craving scores fluctuated irrespective of DBS status, with OCCS from baseline to the end of the sham stimulation fluctuating from 36/56 to 3/56, whilst OCCS from baseline to the end of the DBS-ON fluctuating from 1/56 to 30/56. Urinary cocaine tests confirmed an absence of correlation between crack use and STN-DBS status. Indeed, 3 out of 4 weekly urinary cocaine tests were positive at baseline, SHAM-DBS and ON-DBS periods. We could not enroll the patient in the scheduled open-label ON-DBS follow-up because he decided to move back to his family home, located overseas, where DBS could not be safely monitored. Thus, DBS was turned OFF when he finally reached abstinence and moved to his native hometown. His return to his native hometown was a long-standing plan that had been continually thwarted by a lack of funds due to long-term crack cocaine use.

## Discussion

During this trial, the patient was monitored and intensively treated for his substance use disorder over 24 months consisting of 19 months of inpatient treatment, intensive outpatient treatments, including a dedicated social worker, social housing for 5 months, individual CBT, and pharmacological treatment optimization. He maintained alcohol abstinence for 19 months and showed low-risk alcohol use 5 months afterward. After his return to his native town, 6 months after the end of the cross-over and without STN-DBS, the patient was still abstinent from cocaine (and other stimulants), and maintained an abstinence from buprenorphine and other opioids, as well as benzodiazepines and cyamemazine. The patient viewed the treatment trial as a success. However, the trial failed to demonstrate a significant reduction in both craving and cocaine use under STN-DBS set with standard parameters during the double-blind phase of the protocol. This is parsimonious with the only previous DBS double-blind cross-over trial for cocaine dependence that targeted the Nucleus Accumbens, which also found a long term improvement in the absence of any chronological correlation with the DBS-ON phase (11). Importantly, our trial showed a severe adverse event due to STN-DBS that occurred at parameters previously well tolerated. Hypomania has already been observed in two patients under ventral striatum DBS for methamphetamine dependence (10), and is even presented by the authors as a desirable effect to counteract withdrawal-associated depressive symptoms presented by patients suffering from severe methamphetamine dependence. However, this effect was observed at much high stimulation parameters, namely 150 Hz, 210 μs, and 2.5 V in one patient and 165 Hz, 240 μs, and 3.3 V in the other, and occurred from the initiation of stimulation.

Here the patient experimented hypomania at 130 Hz, 60 μs, and 3 V that he had previously tolerated 6 months earlier. The two main differences between the two challenges were that for the second challenge, the patient had used crack recently and that the tapering of several sedative treatments was ongoing when DBS was reapplied. This highlights a possible interaction between the re-start of the STN-DBS and concomitant cocaine intake, which merits further investigation.

Other stimulation parameters also require further investigation. Recent animal studies suggest that low, but not high, frequency STN-DBS can be effective in reducing cocaine intake in a model of compulsive cocaine intake (14). More detailed exploration of the mechanisms associated with DBS treatment response, as suggested by recent data for several other DBS-treated conditions (22), may better refine the ability of STN-DBS to modify the brain network (ventral striatum, dorsolateral prefrontal cortex and orbitofrontal cortex) associated with cue-induced cocaine craving (23). This can be achieved during laboratory sessions, thereby guiding personalized, tailored, progressive parameter settings. Future trials should include a prolonged abstinence period before starting STN-DBS and include progressive voltage increase, over several weeks, in a carefully monitored environment.

## Conclusion

We failed to demonstrate a significant decrease in cocaine craving in the DBS-ON, vs. DBS-SHAM, double-blind controlled trial of STN-DBS in one patient with refractory crack cocaine dependence. Over the 2-year treatment period, the patient reduced his crack use and finally reached abstinence. However, this was not correlated with DBS-ON periods. STN-DBS for crack–cocaine dependence may be associated with serious impacts on affective state and should be performed only with caution in crack cocaine therapeutic research trials. Our data will prove of benefit to future treatment interventions, including as to a study design that involves a prolonged abstinence period before initiating STN-DBS, a personalized cue-induced cocaine craving brain network monitoring to choose the best target and a progressive voltage increase over several weeks in a carefully monitored environment.

## Data availability statement

The datasets presented in this article are not readily available because the generated data belong to the Clinical Research Direction (DRCI) from the Assistance Publique – Hôpitaux de Paris (APHP) and may be accessible upon reasonable request under the restriction of French laws. Requests to access the datasets should be directed to luc.mallet@inserm.fr.

## Ethics statement

The studies involving human participants were reviewed and approved by the Committee for the Protection of People Ile-de-France VI. The patients/participants provided their written informed consent to participate in this study. Written informed consent was obtained from the participant/patient(s) for the publication of this case report.

## Author contributions

FV and PD wrote the first draft of the manuscript. LM and FV designed the study. FV, PD, CK, HB, DG, and LM collected the data. All authors have actively participated in the manuscript revision and approved the final version.

## References

[1] Degenhardt L Singleton J Calabria B McLaren J Kerr T Mehta S. Mortality among cocaine users: a systematic review of cohort studies. Drug Alcohol Depend. (2011) 113:88–95. 10.1016/j.drugalcdep.2010.07.026 20828942

[2] Degenhardt L Whiteford HA Ferrari AJ Baxter AJ Charlson FJ Hall WD. Global burden of disease attributable to illicit drug use and dependence: findings from the Global Burden of Disease Study 2010. Lancet. (2013) 382:1564–74. 10.1016/S0140-6736(13)61530-5 23993281

[3] Cornish JW O'Brien CP. Crack cocaine abuse: an epidemic with many public health consequences. Annu Rev Public Health. (1996) 17:259–73. 10.1146/annurev.pu.17.050196.001355 8724227

[4] Goulian A Jauffret-Roustide M Dambélé S Singh R Fullilove RE. A cultural and political difference: comparing the racial and social framing of population crack cocaine use between the United States and France. Harm Reduct J. (2022) 19:44. 10.1186/s12954-022-00625-5 35550157 PMC9096779

[5] Toledo L Góngora A Bastos FIPM. On the sidelines of society: crack use, deviation, criminalization and social exclusion - a narrative review. Cien Saude Colet. (2017) 22:31–42. 10.1590/1413-81232017221.02852016 28076527

[6] Antoine J Berndt N Astudillo M Cairns D Jahr S Jones A. Cocaine treatment demands in 10 western European countries: observed trends between 2011 and 2018. Addiction. (2021) 116:1131–43. 10.1111/add.15237 32860458 PMC8247055

[7] Eiden C Vincent M Serrand C Serre A Richard N Picot M. Health consequences of cocaine use in France: data from the French Addictovigilance Network. Fundam Clin Pharmacol. (2020) 35:455–65. 10.1111/fcp.12603 32854152

[8] Schwartz EKC Wolkowicz NR De Aquino JP MacLean RR Sofuoglu M. Cocaine use disorder (CUD): current clinical perspectives. Subst Abuse Rehabil. (2022) 13:25–46. 10.2147/SAR.S337338 36093428 PMC9451050

[9] Brandt L Chao T Comer SD Levin FR. Pharmacotherapeutic strategies for treating cocaine use disorder-what do we have to offer? Addiction. (2021) 116:694–710. 10.1111/add.15242 32888245 PMC7930140

[10] Ge S Chen Y Li N Qu L Li Y Jing J. Deep brain stimulation of nucleus accumbens for methamphetamine addiction: two case reports. World Neurosurg. (2019) 122:512–7. 10.1016/j.wneu.2018.11.056 30448569

[11] Gonçalves-Ferreira A do Couto FS Rainha Campos A Lucas Neto LP Gonçalves-Ferreira D Teixeira J. Deep brain stimulation for refractory cocaine dependence. Biol Psychiatry. (2016) 79:e87–9. 10.1016/j.biopsych.2015.06.023 26235303

[12] Zhang C Wei H Zhang Y Du J Liu W Zhan S. Increased dopamine transporter levels following nucleus accumbens deep brain stimulation in methamphetamine use disorder: a case report. Brain Stimulation. (2019) 12:1055–7. 10.1016/j.brs.2019.02.023 30853339

[13] Chang R Peng J Chen Y Liao H Zhao S Zou J. Deep brain stimulation in drug addiction treatment: research progress and perspective. Front Psychiatry. (2022) 13:858638. 10.3389/fpsyt.2022.858638 35463506 PMC9022905

[14] Vorspan F Mallet L Corvol JC Pelissolo A Lépine JP. Treating addictions with deep brain stimulation is premature but well-controlled clinical trials should be performed. Addiction. (2011) 106:1535–6. 10.1111/j.1360-0443.2011.03450.x 21749519

[15] Rouaud T Lardeux S Panayotis N Paleressompoulle D Cador M Baunez C. Reducing the desire for cocaine with subthalamic nucleus deep brain stimulation. Proc Natl Acad Sci USA. (2010) 107:1196–200. 10.1073/pnas.0908189107 20080543 PMC2824319

[16] Pelloux Y Degoulet M Tiran-Cappello A Cohen C Lardeux S George O. Subthalamic nucleus high frequency stimulation prevents and reverses escalated cocaine use. Mol Psychiatry. (2018) 23:2266–76. 10.1038/s41380-018-0080-y 29880881 PMC8276917

[17] Degoulet M Tiran-Cappello A Combrisson E Baunez C Pelloux Y. Subthalamic low-frequency oscillations predict vulnerability to cocaine addiction. Proc Natl Acad Sci USA. (2021) 118:e2024121118. 10.1073/pnas.2024121118 33785599 PMC8040637

[18] Lhommée E Klinger H Thobois S Schmitt E Ardouin C Bichon A. Subthalamic stimulation in Parkinson's disease: restoring the balance of motivated behaviours. Brain. (2012) 135:1463–77. 10.1093/brain/aws078 22508959

[19] Mallet L Polosan M Jaafari N Baup N Welter ML Fontaine D. Subthalamic nucleus stimulation in severe obsessive-compulsive disorder. N Engl J Med. (2008) 359:2121–34. 10.1056/NEJMoa0708514 19005196

[20] Mallet L Du Montcel ST Clair AH Arbus C Bardinet E Baup N. Long-term effects of subthalamic stimulation in obsessive-compulsive disorder: follow-up of a randomized controlled trial. Brain Stimul. (2019) 12:1080–2. 10.1016/j.brs.2019.04.004 30992192

[21] Polosan M Chabardes S Bougerol T Ardouin C Pollak P Benabid AL. Long-term improvement in obsessions and compulsions with subthalamic stimulation. Neurology. (2016) 87:1843–4. 10.1212/WNL.0000000000003248 27655738

[22] Tyagi H Apergis-Schoute AM Akram H Foltynie T Limousin P Drummond LM. A randomized trial directly comparing ventral capsule and anteromedial subthalamic nucleus stimulation in obsessive-compulsive disorder: clinical and imaging evidence for dissociable effects. Focus. (2022) 20:160–9. 10.1176/appi.focus.20105 35746938 PMC9063594

[23] Skinner MD Aubin HJ. Craving's place in addiction theory: contributions of the major models. Neurosci Biobehav Rev. (2010) 34:606–23. 10.1016/j.neubiorev.2009.11.024 19961872

[24] Vorspan F Brousse G Bloch V Bellais L Romo L Guillem E. Cocaine-induced psychotic symptoms in French cocaine addicts. Psychiatry Res. (2012) 200:1074–6. 10.1016/j.psychres.2012.04.008 22551940

[25] Crum RM Anthony JC. Cocaine use and other suspected risk factors for obsessive-compulsive disorder: a prospective study with data from the Epidemiologic Catchment Area surveys. Drug Alcohol Depend. (1993) 31:281–95. 10.1016/0376-8716(93)90010-N 8462416

[26] Vorspan F Bellais L Romo L Bloch V Neira R Lépine JP. The Obsessive-Compulsive Cocaine Scale (OCCS): a pilot study of a new questionnaire for assessing cocaine craving. Am J Addict. (2012) 21:313–9. 10.1111/j.1521-0391.2012.00248.x 22691009

[27] Sussner BD Smelson DA Rodrigues S Kline A Losonczy M Ziedonis D. The validity and reliability of a brief measure of cocaine craving. Drug Alcohol Depend. (2006) 83:233–7. 10.1016/j.drugalcdep.2005.11.022 16384655

[28] Bardinet E Bhattacharjee M Dormont D Pidoux B Malandain G Schüpbach M. A three-dimensional histological atlas of the human basal ganglia. II. Atlas deformation strategy and evaluation in deep brain stimulation for Parkinson disease: Clinical article. JNS. (2009) 110:208–19. 10.3171/2008.3.17469 18976051

